# Traumatic Oral Lesions in Loggerhead Sea Turtles (*Caretta caretta*) Linked to Polychaete (*Laetmonice* cf. *hystrix*) Ingestion: A Case Report from the Northern Adriatic Sea

**DOI:** 10.3390/ani15182727

**Published:** 2025-09-18

**Authors:** Stefano Pesaro, Lucia Biagini, Danilo De Bellis, Luca Dorigo, Alice Baggio, Isabella Perlin, Giacomo Rossi

**Affiliations:** 1 Department of Agricultural, Food, Environmental and Animal Sciences, University of Udine, 33100 Udine, Italy; stefano.pesaro@uniud.it (S.P.); alice.baggio@uniud.it (A.B.); isabella.perlin@uniud.it (I.P.); 2 School of Biosciences and Veterinary Medicine, University of Camerino, 62024 Matelica, Italy; lucia.biagini@unicam.it (L.B.); giacomo.rossi@unicam.it (G.R.); 3 Friulian Museum of Natural History, 33100 Udine, Italy; luca.dorigo1979@gmail.com

**Keywords:** sea turtle, *Caretta caretta*, oral lesion, polychaete, wildlife, pathology

## Abstract

Oral cavity lesions in sea turtles, particularly *Caretta caretta*, are relatively rare, and are typically linked to infectious agents as well as anthropogenic factors, including ingestion of marine debris or fishing gear. This report describes a juvenile *Caretta caretta* found in the northern Adriatic Sea with severe oral lesions affecting the choanae, alimentary tract, and larynx. A comprehensive clinical and histopathological evaluation was conducted, which revealed traumatic injuries caused by the ingestion of a polychaete, *Laetmonice* cf. *hystrix.* Mucosal biopsies in the areas of spine penetration revealed the presence of strong sub-epithelial inflammation, characterised by micro-abscesses. In addition, around some fragments of spines, the formation of microgranulomatous lesions with a tendency to encapsulation was observed. Treatment protocol involved the removal of embedded spines and administration of a broad-spectrum antibiotic to prevent secondary infections. Recently, the detection in polychaetes of the aetiological agents of two newly emerging diseases of shrimps, suggests that these worms can be a host or/and passive carrier of these pathogens. This case study underscores the necessity to consider both biological and anthropogenic factors in diagnosing and managing oral lesions in marine turtles. Furthermore, it draws attention to the ecological risks posed by interactions between sea turtles and benthic organisms.

## Simple Summary

This case report describes a rare clinical finding of traumatic oral lesions in a juvenile loggerhead sea turtle (*Caretta caretta*) rescued from the Northern Adriatic Sea. The turtle exhibited severe injuries in the oral cavity, upper gastrointestinal tract, and larynx, caused by the accidental ingestion of a tropical benthic polychaete, *Laetmonice* cf. *hystrix*. The worm’s harpoon-shaped chaetae had penetrated the mucosal tissues, leading to subepithelial inflammation, microabscesses, and granulomatous reactions. Histological analysis revealed marked hyperkeratosis, dyskeratosis, and encapsulated spicule fragments surrounded by pyogranulomatous inflammation. Treatment included careful removal of the spines and administration of a broad-spectrum antibiotic. This report highlights the importance of considering biological causes, in addition to anthropogenic causes, when diagnosing oral lesions in sea turtles. The unusual ingestion of this polychaete may be linked to shifting prey availability due to climate change and reduced fish stocks, potentially altering turtle foraging behaviour. Furthermore, recent studies suggest that polychaetes can act as carriers of emerging shrimp pathogens, raising ecological and health concerns. This case underlines the need for further research into the role of benthic organisms in sea turtle health and marine trophic networks, especially under the influence of environmental changes.

## 1. Introduction

The loggerhead sea turtle (*Caretta caretta*) is a species that is distributed across the globe and exhibits migratory behaviour. The International Union for Conservation of Nature (IUCN) Red List of Threatened Species, classifies the loggerhead sea turtle (*Caretta caretta*) as “Least Concern” within the mediterranean bio-region. This classification is attributed to the decades of intensive conservation programmes that have been implemented [1,2]. As is the case in other geographical areas, the conservation of this species in the Mediterranean region is of critical importance. This necessity arises from the requirement for comprehensive knowledge of essential biological aspects, including reproductive biology, migratory patterns, and feeding ecology [3,4]. As is well documented, loggerhead sea turtles are carnivorous throughout their life cycle, with fish representing the primary dietary component, followed by pelagic tunicates, crustaceans, molluscs, and various other invertebrates such as polychaetas [5,6,7]. Upper and lower gastrointestinal disorders are recognised as a significant cause of sea turtle disease on a global scale. A high prevalence of these disorders has been observed across diverse populations and various life stages, ranging from post-hatching to adults [8,9,10,11,12]. Gastrointestinal abnormalities characteristically affect regions ranging from the oesophagus to the intestinal tract. The aetiology is frequently the ingestion of anthropogenic debris, microbial infections, intestinal volvulus, or intestinal impaction [13,14,15,16]. Documented cases of lesions affecting the upper gastrointestinal tract of sea turtles, with a particular focus on those affecting the oral cavity of loggerhead sea turtles, have been reported. The predominant characteristics of these lesions are traumatic in nature, primarily resulting from the ingestion of fish hooks [17]. However, it should be noted that the condition may also encompass infectious forms, such as bacterial or viral ulcerative stomatitis and glossitis, as well as proliferative forms, including oral fibropapillomatosis [11,15,18,19], though such cases are rare.

The objective of this study is to provide the first description of oral cavity abnormalities observed in a juvenile *Caretta caretta* rescued from the Northern Adriatic Sea, presenting a detailed macroscopic and microscopic characterisation of lesions associated with ingestion of the tropical benthic polychaete, *Laetmonice* cf. *hystrix*.

## 2. Case Presentation

### 2.1. Rescue and Clinical Findings

A loggerhead sea turtle classified as a pelagic juvenile basing on the work of previous authors [20,21] was found in July 2024 in the Northern Adriatic Sea, in the vicinity of the coast near the town of Grado (45°40′ N, 13°23′ W). The specimen exhibited slow and poorly responsive swimming behaviour. The animal was rescued and transferred to the San Canzian d’Isonzo (Gorizia) rescue centre (Centro Regionale Recupero Fauna Esotica, Selvatica e Tartarughe Marine) where a comprehensive medical examination was conducted.

**Figure 1:**
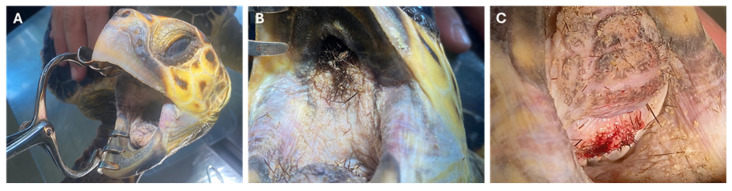
Image of the rescued subject (**A**) and gross appearance of the filamentous structures adherent to the oral mucosal, obstructing the oral lumen (**B**) and the proximal oesophagus (**C**).

The turtle presented a straight carapace length (SCL) of 40 cm, a straight carapace width (SCW) of 36 cm and a body weight of 7 kg. A thorough clinical examination of the animal was conducted, revealing that, while it appeared to be vigorous, it exhibited a distinctive breathing pattern characterised by open-mouth breathing. Barnacles were observed attached to both the carapace and the plastron surfaces. No additional pathological or behavioural abnormalities were detected during the general physical examination. A detailed oral cavity examination was performed to assess the condition of the beak, oral mucosa, and tongue. This examination revealed numerous rigid, brown, filamentous structures firmly adhered to the oral mucosa, which nearly completely obstructed the choanal lumen and were particularly concentrated along the ventral margin and base of the tongue (Figure 1). A smaller number of similar structures were detected in the proximal oesophagus, interspersed among the papillae. The presence of mild to severe hyperkeratosis was observed across the oral mucosa, including the tongue. The laryngeal region exhibited a narrowed lumen and prominent keratinized mucosal folds (Figure 1C).

The clinical and biometric data of the specimen have been entered into the wildlife monitoring and surveillance data repository system InfoFaunaFGV [22].

### 2.2. Diagnostic Tests

**Figure 2:**
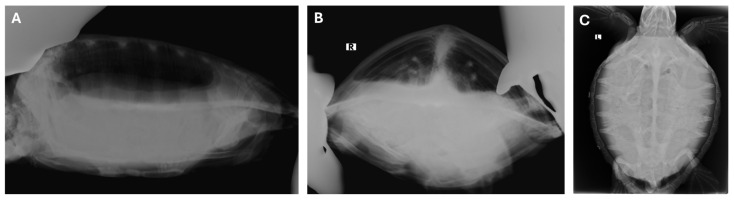
Radiographic projections. (**A**) Latero-lateral and (**B)** craniocaudal projections demonstrate a mild increase in pulmonary radiopacity, especially on the right region. (**C**) Dorsoventral projection is included for anatomical reference.

In order to comprehensively evaluate the animal’s health status, a blood sample (a minimum of 1 mL) was extracted from the dorsal cervical sinus, which is derived from the post-occipital venous plexus, and placed into lithium-heparin tubes after surgical preparation of the site. The haematocrit value was obtained by means of standard centrifugation in microhematocrit capillary tubes. The biochemical values were obtained using a Vetscan VS2 analyser (Abaxis Inc., Union City, CA 94587, USA). A comparison of the blood test results with the reference values established by Deem et al. (2009) and Teare JA (2013) [23,24], was conducted and revealed no significant abnormalities. A radiographic assessment was conducted in latero-lateral, cranio-caudal, and dorso-ventral projection (Figure 2A–C). This revealed a mild increase in radiopacity localised in the cranioventral region of the right lung.

### 2.3. Identification of Foreign Bodies and Histological Analysis

**Figure 3:**
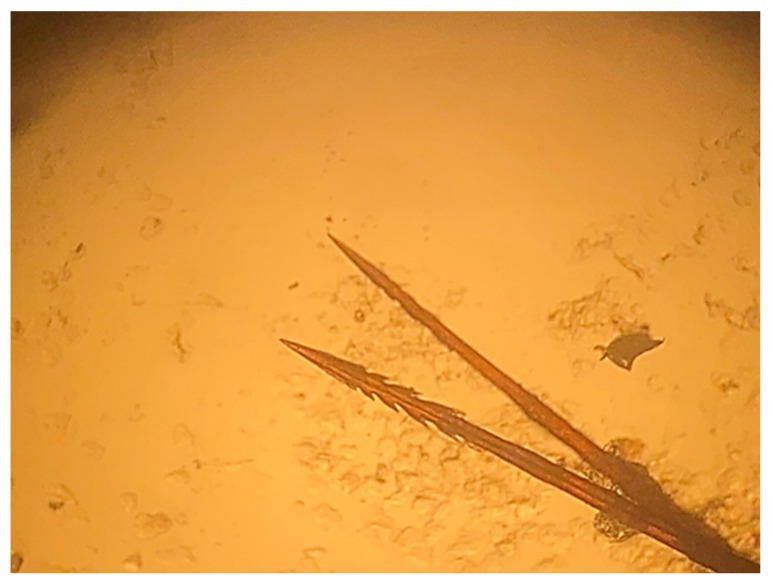
Harpoon-shaped notochaetae extracted from the oral cavity of the *Caretta caretta* specimen.

In order to facilitate feeding and respiratory functions, as well as to determine the nature of the embedded structures and assess tissue damage, the filamentous elements were carefully removed and examined under a stereomicroscope at the Friulan museum of natural history. Furthermore, mucosal biopsies were collected in the proximity of the laryngeal region for the purpose of histopathological analysis. Bioptic samples were fixed in 10% buffered formalin and dispatched to the animal pathology laboratory of the School of Biosciences and Veterinary Medicine of the University of Camerino where they were routinely processed for histological analysis [25]. Briefly, biopsies were embedded in paraffin and sectioned at 3 µm for haematoxylin and eosin (HE) staining. Sections were mounted on positively charged glass slides (Superfrost Plus, Fisher, Pittsburgh, PA, USA) for microscopic evaluation. The morphological evaluation of the foreign bodies revealed them to be harpoon-shaped notochaetae. These characteristics are indicative of the genus *Laetmonice* Kinberg, 1856, within the polychaete family *Aphroditidae.* The identification of the foreign bodies was confirmed as *Laetmonice* cf. *hystrix* (Figure 3) [26]. These elongated, pointed, latero-dorsal chaetae are located along the dorsum but have the capacity to be erected in response to physical stimulation, thus serving an active defensive function against predators. The presence of this distinctive type of chaeta within the oral cavity of *Caretta caretta* facilitated the identification of the polychaete [27,28,29].

**Figure 4:**
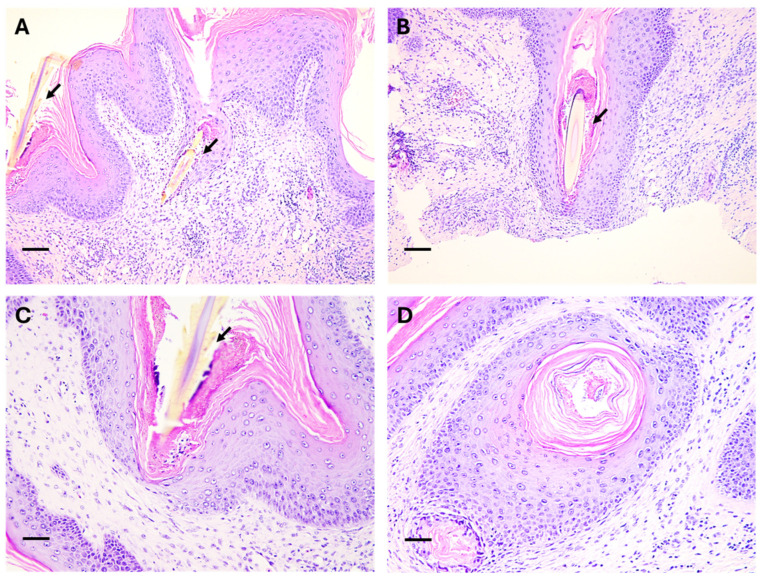
Histological aspects of the biopsies obtained from the oral mucosa of the loggerhead sea turtles (*Caretta caretta*). (**A**,**B**) Various spiculae of polychaetae identified as *Laetmonice* cf. *hystrix* penetrating the mucosal epithelium are observed (**Arrows**), and associated with hyperkeratosis, dyskeratosis and heterophilic inflammation. (**C**) Higer magnification of a spicula penetrating the mucosa with associated inflammation, and (**D**) hyperkeratosis associated with accumulations of amorphous keratin within epidermal folds. H&E stain; scale bars = 200 µm (**A**,**B**); 100 µm (**C**,**D**).

A detailed histopathological examination revealed significant hyperkeratosis and dyskeratosis, with accumulations of amorphous keratin within epidermal folds (Figure 4). In the submucosal layer, there was a marked focal heterophilic inflammation, which was characterised by the presence of microabscess around the spicular penetration sites. Pyogranulomatous reactions were evident around several embedded spines, and these were characterised by granuloma formation encapsulating fragments of spicular remnants. The final diagnosis is of chronic active mucositis, with microgranulomatous areas surrounding portions of parasitic spicules. The peri-spiculate reaction is characterised by the manifestation of a foreign body reaction.

### 2.4. Treatments and Outcome

As previously stated, the spines were extracted from the oral cavity and choanae to facilitate the animal’s ability to feed and breathe through its nostrils. Following the removal of the spines, the animal was treated with broad-spectrum antibiotics (enrofloxacin (5 mg/kg, q48 h, IM) for 5 days [30]) to address any potential infections caused by pathogens entering through the foreign bodies. The animal was successfully released into the open sea after seven days, once it had been determined that it was capable of feeding independently (a capacity that was absent in the first few days following the removal of the spines), that it had excellent diving abilities, and that it did not breathe through its mouth.

## 3. Discussion

Although pathological alterations of the upper alimentary tract have previously been reported in loggerhead sea turtles (*Caretta caretta*), these have been predominantly associated with the ingestion of anthropogenic foreign bodies, particularly fishing hooks, marine debris, or with bacterial and viral etiologies [9,11,15,17,18,31]. However, a case of improper feeding behaviour associated with gastric perforation after ingestion of a sharptail eel (*Myrichthys ocellatus*) has been previously reported [32]. To date, however, no descriptions have been provided of chronic-active mucositis characterised by microgranulomatous lesions resulting from inappropriate predatory and feeding behaviour. *Laetmonice* cf. *hystrix* is a species of polychaete worm that inhabits the benthic zone. It belongs to the family *Aphroditidae*, which is more commonly referred to as ‘scale worms’ or ‘sea mice’. Its distribution encompasses the Western Atlantic, the Mediterranean Sea, and the Indo-West Pacific, with a preference for tropical to subtropical regions. The consumption of polychaetes by *Caretta caretta*, although previously reported as part of the species’ diet in Mediterranean populations [33], remains poorly documented and difficult to investigate. Notably, the ingestion of *Laetmonice* cf. *hystrix* has not been previously documented in the scientific literature. In light of the extant literature, the hypothesis concerning the ecological pressures that may have driven this improper feeding behaviour remains entirely speculative. Possible explanations include regional variation in benthic prey availability in the Mediterranean [34], changes in the population dynamics of polychaetes [35], or simply opportunistic feeding behaviour of loggerhead sea turtles [7].

It has been determined that climate change is a substantial contributing factor to the observed alterations in marine ecosystems. These alterations encompass, but are not limited to, the reduction in native species in certain areas and the distribution of new species, whether invasive or native, that expand their habitats due to changing trophic resources or rising water temperatures that create favourable conditions for colonising new areas [36,37]. The decline of small pelagic fishes and demersal fish species in the Northern Adriatic Sea [37] may have prompted predatory species, such as sea turtles, to modify their dietary patterns. This modification may have resulted in the identification of novel food sources and the consumption of species that are less frequently targeted, such as *Laetmonice* cf. *hystrix*. These species have been documented as being more prevalent in the southern Mediterranean region [38,39]; however, there is a possibility that they may also be found in northern regions due to the impact of rising sea water temperatures. The Adriatic Sea is well recognised as one of the main foraging areas for marine turtles in the Mediterranean Sea [40]. The introduction of novel, invasive species, such as the *Laetmonice* cf. *hystrix* has the potential to constitute a novel threat to sea turtles, particularly in instances where their customary prey species are absent. Recent studies have reported the detection of the aetiological agents of two emerging shrimp diseases, namely *Enterocytozoon hepatopenaei* (EHP) and *Vibrio parahaemolyticus*, in polychaetes [41]. This further suggests that these worms may play a role as hosts and/or passive carriers of pathogens, a hypothesis which requires further investigation.

## 4. Conclusions

This report constitutes the first comprehensive account of the pathological feeding interaction between loggerhead sea turtles and the polychaete species *Laetmonice* cf. *hystrix*. Recently, analogous cases have been detected by colleagues on specimens from the Northern Adriatic Sea. However, due to a lack of proper investigation, these have not been considered in the present report. In view of the increase in the number of reports, the authors will focus on investigating any future cases that may arise, with a view to describing the impact of this new species on the health of the Adriatic Sea turtle population in the future. In fact, although this study demonstrates the direct traumatic effects of *Laetmonice* cf. *hystrix* consumption on the oral cavity, other causes should be considered in the differential diagnosis of stomatitis, not attributable to anthropogenic or microbiological causes. The present findings emphasise the necessity for further investigation to be conducted into the ecological and pathological roles of polychaetes in the trophic networks of marine ecosystems, with particular reference to vulnerable species such as sea turtles.

## Data Availability

All data relating to and supporting this case report are available by logging in to the InfoFaunaFVG web app at the link https://infofaunafvg.com/cras/web/app.php/login (accessed on 3 August 2024).

## Abbreviations

The following abbreviations are used in this manuscript:

SCL	Straight carapace length
SCW	Straight carapace width
HE	Haematoxylin Eosin
IM	Intra-Muscular

## References

[1] Casale P. (2015). The IUCN Red List of Threatened Species: *Caretta caretta* Mediterranean Subpopulation. https://www.iucnredlist.org/species/83644804/83646294.

[2] Zampollo A., Arcangeli A., Costantino M., Mancino C., Crosti R., Pietroluongo G., Giacoma C., Azzolin M. (2022). Seasonal Niche and Spatial Distribution Modelling of the Loggerhead (*Caretta caretta*) in the Adriatic and Ionian Seas. Aquat. Conserv. Mar. Freshw. Ecosyst..

[3] Burke V.J., Standora E.A., Morreale S.J. (1993). Diet of Juvenile Kemp’s Ridley and Loggerhead Sea Turtles from Long Island, New York. Copeia.

[4] Casale P., Broderick A., Camiñas J., Cardona L., Carreras C., Demetropoulos A., Fuller W., Godley B., Hochscheid S., Kaska Y. (2018). Mediterranean Sea Turtles: Current Knowledge and Priorities for Conservation and Research. Endanger. Species Res..

[5] Tomas J., Aznar F.J., Raga J.A. (2001). Feeding Ecology of the Loggerhead Turtle *Caretta caretta* in the Western Mediterranean. J. Zool..

[6] Revelles M., Cardona L., Aguilar A., Fernández G. (2007). The Diet of Pelagic Loggerhead Sea Turtles (*Caretta caretta*) off the Balearic Archipelago (Western Mediterranean): Relevance of Long-Line Baits. J. Mar. Biol. Assoc. UK.

[7] Mariani G., Bellucci F., Cocumelli C., Raso C., Hochscheid S., Roncari C., Nerone E., Recchi S., Di Giacinto F., Olivieri V. (2023). Dietary Preferences of Loggerhead Sea Turtles (*Caretta caretta*) in Two Mediterranean Feeding Grounds: Does Prey Selection Change with Habitat Use throughout Their Life Cycle?. Animals.

[8] Glazebrook J.S., Campbell R.S.F. (1990). A Survey of the Diseases of Marine Turtles in Northern Australia. I. Farmed Turtles. Dis. Aquat. Org..

[9] Glazebrook J.S., Campbell R.S.F. (1990). A Survey of the Diseases of Marine Turtles in Northern Australia. II. Oceanarium-Reareo and Wild Turtles. Dis. Aquat. Org..

[10] Orós J., Torrent A., Calabuig P., Déniz S. (2005). Diseases and Causes of Mortality among Sea Turtles Stranded in the Canary Islands, Spain (1998–2001). Dis. Aquat. Org..

[11] Orós J., Déniz S., Calabuig P. (2004). Digestive Pathology of Sea Turtles Stranded in the Canary Islands between 1993 and 2001. Vet. Rec..

[12] Orós J., Montesdeoca N., Camacho M., Arencibia A., Calabuig P. (2016). Causes of Stranding and Mortality, and Final Disposition of Loggerhead Sea Turtles (*Caretta caretta*) Admitted to a Wildlife Rehabilitation Center in Gran Canaria Island, Spain (1998–2014): A Long-Term Retrospective Study. PLoS ONE.

[13] George R.H. (1997). Health Problems and Diseases of Sea Turtles. The Biology of Sea Turtles, Volume I.

[14] Torrent A., Déniz S., Ruiz A., Calabuig P., Sicilia J., Orós J. (2002). Esophageal Diverticulum Associated with *Aerococcus viridans* Infection in a Loggerhead Sea Turtle (*Caretta caretta*). J. Wildl. Dis..

[15] Stacy B.A., Wellehan J.F.X., Foley A.M., Coberley S.S., Herbst L.H., Manire C.A., Garner M.M., Brookins M.D., Childress A.L., Jacobson E.R. (2008). Two Herpesviruses Associated with Disease in Wild Atlantic Loggerhead Sea Turtles (*Caretta caretta*). Vet. Microbiol..

[16] Caracappa S., Persichetti M.F., Piazza A., Caracappa G., Gentile A., Marineo S., Crucitti D., Arculeo M. (2018). Incidental Catch of Loggerhead Sea Turtles (*Caretta caretta*) along the Sicilian Coasts by Longline Fishery. PeerJ.

[17] Parga M.L. (2012). Hooks and Sea Turtles: A Veterinarian’s Perspective. Bull. Mar. Sci..

[18] Glazebrook J.S., Campbell R.S.F., Thomas A.T. (1993). Studies on an Ulcerative Stomatitis-Obstructive Rhinitis-Pneumonia Disease Complex in Hatching and Juvenile Sea Turtles *Chelonia mydas* and *Caretta caretta*. Dis. Aquat. Org..

[19] Aguirre A.A., Balazs G.H., Spraker T.R., Murakawa S.K.K., Zimmerman B. (2002). Pathology of Oropharyngeal Fibropapillomatosis in Green Turtles *Chelonia mydas*. J. Aquat. Anim. Health.

[20] Bjorndal K., Bolten A., Martins H. (2000). Somatic Growth Model of Juvenile Loggerhead Sea Turtles *Caretta caretta*: Duration of Pelagic Stage. Mar. Ecol. Prog. Ser..

[21] Casal A.B., Camacho M., López-Jurado L.F., Juste C., Orós J. (2009). Comparative Study of Hematologic and Plasma Biochemical Variables in Eastern Atlantic Juvenile and Adult Nesting Loggerhead Sea Turtles (*Caretta caretta*). Vet. Clin. Pathol..

[22] Tomè P., Pesaro S., Orioles M., Pascotto E., Cadamuro A., Galeotti M. (2023). InfoFaunaFVG: A Novel Progressive Web Application for Wildlife Surveillance. Eur. J. Wildl. Res..

[23] Deem S.L., Norton T.M., Mitchell M., Segars A., Alleman A.R., Cray C., Poppenga R.H., Dodd M., Karesh W.B. (2009). Comparison of Blood Values in Foraging, Nesting, and Stranded Loggerhead Turtles (*Caretta caretta*) Along the Coast of Georgia, USA. J. Wildl. Dis..

[24] Teare J.A. (2013). Species360 Physiological Reference Intervals for Captive Wildlife.

[25] Desantis S., Galosi L., Santamaria N., Roncarati A., Biagini L., Rossi G. (2021). Modulation of Morphology and Glycan Composition of Mucins in Farmed Guinea Fowl (*Numida meleagris*) Intestine by the Multi-Strain Probiotic Slab51^®^. Animals.

[26] Wu X., Hutchings P., Murray A., Xu K. (2021). *Laetmonice iocasica* Sp. Nov., a New Polychaete Species (Annelida: Aphroditidae) from Seamounts in the Tropical Western Pacific, with Remarks on *L. producta* Grube, 1877. J. Oceanol. Limnol..

[27] Parapar J., Moreira J., Gambi M.C., Caramelo C. (2013). Morphology and Biology of *Laetmonice producta producta* Grube (Polychaeta: Aphroditidae) in the Bellingshausen Sea and Antarctic Peninsula (Southern Ocean, Antarctica). Ital. J. Zool..

[28] Barnich R. (2003). The Aphroditoidea (Annelida, Polychaeta) of the Mediterranean Sea. Abh. Senckenberg. Naturforschenden Ges..

[29] Pleijel F. (1991). Polychaetes: British Phyllodocoideans, Typhloscolecoideans and Tomopteroideans. Synop. Br. Fauna N S.

[30] Lai O.R., Marín P., Laricchiuta P., Marzano G., Crescenzo G., Escudero E. (2009). Pharmacokinetics of Marbofloxacin in Loggerhead Sea Turtles (*Caretta caretta*) after Single Intravenous and Intramuscular Doses. J. Zoo Wildl. Med..

[31] Casale P., Affronte M., Insacco G., Freggi D., Vallini C., Pino d’Astore P., Basso R., Paolillo G., Abbate G., Argano R. (2010). Sea Turtle Strandings Reveal High Anthropogenic Mortality in Italian Waters. Aquat. Conserv. Mar. Freshw. Ecosyst..

[32] Oliveira R.E.M., Pires J.M.L., Batista J.S., Attademo F.L.N., Farias D.d., Freire A.C.B., Bomfim A.D.C., Lima L.R.P., Oliveira R.M., Gavilan S.A. (2020). Death of a Loggerhead Sea Turtle (*Caretta caretta*) from Ingestion of an Eel (*Myrichthys ocellatus*). Vet. Med..

[33] Lazar B., Zavodnik D., Grbac I., Tvrtković N. Diet Composition of the Loggerhead Sea Turtle, *Caretta caretta*, in the Northern Adriatic Sea: A Preliminary Study. Proceedings of the 20th Annual Symposium on Sea Turtle Biology and Conservation (NMFS-SEFSC-477).

[34] Patel S.H., Morreale S.J., Panagopoulou A., Bailey H., Robinson N.J., Paladino F.V., Margaritoulis D., Spotila J.R. (2015). Changepoint Analysis: A New Approach for Revealing Animal Movements and Behaviors from Satellite Telemetry Data. Ecosphere.

[35] Sardá R., Pinedo S., Dueso A. Estimating Secondary Production in Natural Populations of Polychaetes: Some General Constraints. https://www.ingentaconnect.com/contentone/umrsmas/bullmar/2000/00000067/00000001/art00037.

[36] Bianchi C.N., Morri C., Chiantore M., Montefalcone M., Parravicini V., Rovere A. (2012). Mediterranean Sea Biodiversity between the Legacy from the Past and a Future of Change. Life Mediterr. Sea Look Habitat Changes.

[37] Giani M., Djakovac T., Degobbis D., Cozzi S., Solidoro C., Umani S.F. (2012). Recent Changes in the Marine Ecosystems of the Northern Adriatic Sea. Estuar. Coast. Shelf Sci..

[38] Çinar M. (2005). Polychaetes from the Coast of Northern Cyprus (Eastern Mediterranean Sea), with Two New Records for the Mediterranean Sea. Cah. Biol. Mar..

[39] Matarrese A., Mastrototaro F., D’onghia G., Maiorano P., Tursi A. (2004). Mapping of the Benthic Communities in the Taranto Seas Using Side-Scan Sonar and an Underwater Video Camera. Chem. Ecol..

[40] Mencacci R., Aiudi L., Angelini V., Casale P., Cerritelli G., Lombardi Moraes K., Pari S., Luschi P. (2023). Satellite Tracking Identifies Important Foraging Areas for Loggerhead Turtles Frequenting the Adriatic Sea, Central Mediterranean. Mediterr. Mar. Sci..

[41] Desrina J.V., Verdegem M.C.J., Vlak J.M. (2018). Polychaetes as Potential Risks for Shrimp Pathogen Transmission. Asian Fish. Sci..

